# Impartially Validated Multiple Deep-Chain Models to Detect COVID-19 in Chest X-ray Using Latent Space Radiomics

**DOI:** 10.3390/jcm10143100

**Published:** 2021-07-14

**Authors:** Bardia Yousefi, Satoru Kawakita, Arya Amini, Hamed Akbari, Shailesh M. Advani, Moulay Akhloufi, Xavier P. V. Maldague, Samad Ahadian

**Affiliations:** 1 Department of Electrical and Computer Engineering, Laval University, Quebec City, QC G1V 0A6, Canada; 2 Terasaki Institute for Biomedical Innovation, Los Angeles, CA 90024, USA; skawakita@terasaki.org (S.K.); sadvani@terasaki.org (S.M.A.); 3 Department of Radiation Oncology, City of Hope Comprehensive Cancer Center, Duarte, CA 91010, USA; aamini@coh.org; 4 Department of Radiology, University of Pennsylvania, Philadelphia, PA 19104, USA; AkbariHA@upenn.edu; 5 Department of Computer Science, Perception Robotics and Intelligent Machines (PRIME) Research Group, University of Moncton, New Brunswick, NB E1A 3E9, Canada; moulay.akhloufi@umoncton.ca

**Keywords:** COVID-19 detection, deep convolutional autoencoder (ConvAE), 2D U-Net model, imaging biomarker, deep-learning features, deep latent space radiomics, chest X-ray imaging

## Abstract

The COVID-19 pandemic continues to spread globally at a rapid pace, and its rapid detection remains a challenge due to its rapid infectivity and limited testing availability. One of the simply available imaging modalities in clinical routine involves chest X-ray (CXR), which is often used for diagnostic purposes. Here, we proposed a computer-aided detection of COVID-19 in CXR imaging using deep and conventional radiomic features. First, we used a 2D U-Net model to segment the lung lobes. Then, we extracted deep latent space radiomics by applying deep convolutional autoencoder (ConvAE) with internal dense layers to extract low-dimensional deep radiomics. We used Johnson–Lindenstrauss (JL) lemma, Laplacian scoring (LS), and principal component analysis (PCA) to reduce dimensionality in conventional radiomics. The generated low-dimensional deep and conventional radiomics were integrated to classify COVID-19 from pneumonia and healthy patients. We used 704 CXR images for training the entire model (i.e., U-Net, ConvAE, and feature selection in conventional radiomics). Afterward, we independently validated the whole system using a study cohort of 1597 cases. We trained and tested a random forest model for detecting COVID-19 cases through multivariate binary-class and multiclass classification. The maximal (full multivariate) model using a combination of the two radiomic groups yields performance in classification cross-validated accuracy of 72.6% (69.4–74.4%) for multiclass and 89.6% (88.4–90.7%) for binary-class classification.

## 1. Introduction

The global pandemic associated with COVID-19 continues to spread across the world. It has led to more than 151 million cases and 3.17 million deaths as of 30 April 2021, according to the World Health Organization (WHO) statistics [1]. The WHO declared this to be a Public Health Emergency of International Concern (PHEIC) on 30 January 2020, and finally, on 11 March 2020, the situation was recognized as a global pandemic [2,3]. The highly contagious nature of this virus, leading to infections similar to the severe acquired respiratory syndrome, increased the importance of early detection of COVID-19 to prevent the further spreading of this disease. In clinical routine, simply available imaging modalities, for instance, chest X-ray (CXR) and thoracic computed tomography (CT) offer significant aid to clinicians for imaging diagnosis [4,5,6,7,8,9,10,11,12,13]. However, the current gold standard is the reverse transcription–polymerase chain reaction (RT-PCR) to diagnose COVID-19 viral infection. However, RT-PCR, which relies upon nasopharyngeal or throat swabs, can be influenced by low viral load or sampling errors [6,10] and thus presents a significant challenge, particularly during the early stage of infection with low sensitivity [11,12,13].

Machine learning, as part of artificial intelligence (AI), has unequivocally revolutionized modern medicine, significantly contributed to the medical imaging field, and actively shown its strength for the battle against COVID-19 by enabling more accurate, safe, reliable, and efficient solutions [14]. Several machine-learning-based pipelines are offered for COVID-19, which aim to automatically segment the lung lobes [15,16,17,18,19,20,21,22,23,24] or provide diagnosis and clinical assessment [7,9,25,26], supporting the unequivocal performance of AI in medical imaging. These approaches often used pretrained deep models or a variety of these models or used conventional imaging biomarkers, which are concerned with the accuracy of the models. However, here, we challenge such analysis by our own designed models to extract deep-imaging throughputs and by independently valid models; all models used in this study to prove the reproducibility of these models.

CT and CXR are two widely used imaging modalities to diagnose or screen for COVID-19 [4,5,6,7,8,9]. Many studies focus on classifying COVID-19 patients from those with other non-COVID-19-related diseases, such as pneumonia [22,23,24,25,26,27,28]. Indeed, there is some degree of similarity between pneumonia and COVID-19 for radiological examinations. Chen et al. [22] proposed a U-Net++-based approach to discriminate COVID-19 versus non-COVID-19 patients on the segmented lung area with 106 chest CT images (51 COVID-19 patients). X-ray imaging modalities are less sensitive than CT imaging [27] while considering the first-line investigation method for COVID-19 screening. However, considering the overwhelming number of COVID-19 cases, routine CT imaging places an immense load on different radiology locations, increasing the necessity of employing CXR imaging to detect COVID-19. To differentiate COVID-19 from pneumonia and healthy cases, Ghoshal et al. [25] presented the Bayesian convolutional neural networks trained on 70 lung CXR images from available data sources with a detection accuracy of 92.9% [28]. This outperformed the standard VGG16 model-based diagnosis, which showed 85.7% detection accuracy.

A comparative analysis on three deep-learning models (i.e., InceptionV3, ResNet50, and combined Inception-ResNetV2) used to detect COVID-19 showed that the ResNet50 model achieved the highest classification accuracy, followed by InceptionV3 and Inception-ResNetV2 while testing on publicly available CXR images [7]. Another ResNet-based COVID-19 detection model obtained an area under the curve (AUC) performance of 95.2% in 1078 X-ray images (1008 non-COVID-19 pneumonia and 70 COVID-19 patients) [26]. Heidari et al. [27] proposed a convolutional neural network (CNN) model through transfer learning to categorize CXR images into the classes of infected pneumonia, COVID-19, other community-acquired no-COVID-19 pneumonia, and normal cases using 8474 diagnostic images and yielded an accuracy of 94.5%. In summary, CNN-based models to detect COVID-19 using CXR imaging include but are not limited to ResNet18 [28,29,30], ResNet50 [7,30,31], MobileNetV2 [8], CoroNet [32], Xception [33], Xception + ResNet50V2 [34], DarkCovidNet [35], COVID-Net [9], COVIDX-Net [36], Parallel-Dilated COVIDNet (PDCOVIDNet) [37], and Deep-COVID [38]. Many of these methods rather used pretrained models to tackle the classification problem or extracting features to be used with the classifier or did not validate their model independently.

This study scrutinizes a hybrid machine learning approach involving a hierarchy of models to computerize the diagnosis of COVID-19 from CXR images. We first applied an intelligent system for automatic segmentation of the lung lobes using U-Net and then used the models trained with conventional and/or deep radiomics to perform assessments on several independent validation sets. U-Net is independently used for our testing datasets after being trained by our training set, which makes it more interesting than previously presented approaches (e.g., [28]). Our study investigates the plausibility of deep and conventional radiomics as imaging biomarkers for diagnosing COVID-19 using CXR. Here, we summarized the contributions of this study as follows:

- The proposed approach trains and independently uses a 2D U-Net model for segmenting the lung lobes in CXR images;
- We proposed a convolutional deep autoencoder (ConvAE) to extract low-dimensional deep-imaging features, called deep radiomics, from CXR images as potential diagnostic biomarkers for COVID-19;
- Our study addresses the curse of dimensionality problem using high-dimensional deep radiomics by utilizing a ConvAE to compress the feature space and combine them with conventional radiomics for diagnostic purposes;
- The proposed model successfully classifies subjects into healthy, pneumonia, and COVID-19 cases through binary- and multiclass classification, as validated with an independent cohort of patients. We also provided comparative analyses of the different combinations of feature categories.

In the next section, the methodology and application of U-Net and ConvAE to segment lung lobes (https://www.kaggle.com/nikhilpandey360/chest-xray-masks-and-labels, accessed on 21 January 2019) and extract deep radiomics are presented. Section 3 and Section 4 show experimental results and discussion, respectively. Section 5 concludes the study with a summary of the results and overall performance.

## 2. Methods

**Figure 1:**
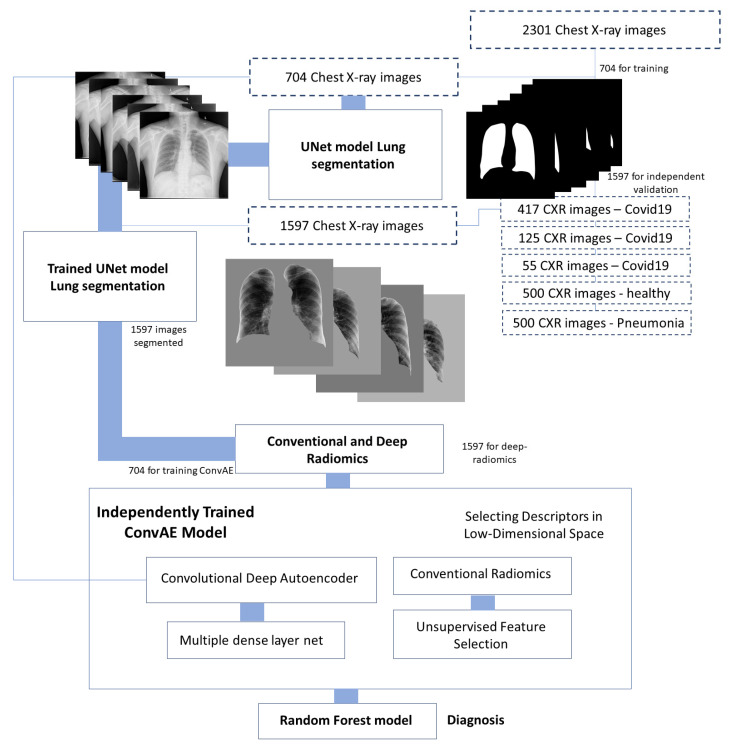
Schematic representing the proposed approach using ConvAE radiomics in low-dimensional space representation for diagnosis of COVID-19.

The proposed methodology relies on a hierarchy of deep learning models for segmenting lung lobes, extracting deep radiomics, and detecting COVID-19 cases from extracted features. The workflow of the proposed study is shown in Figure 1.

### 2.1. U-Net for Segmentation of Lung Lobes

Lung lobes segmentation has achieved reasonable accuracy using different configurations of deep neural networks [15,16,17,18,19,20,21,22,23]. Particularly, automatic segmentation of lung lobes to diagnose COVID-19 uses deep-learning-based models. Among many structures, the U-Net architecture has achieved exceptional capability in segmenting lung images in CT and CXR modalities [15,21]. In this study, we used 2D U-Net to segment lung lobes in CXR images.

**Figure 2:**
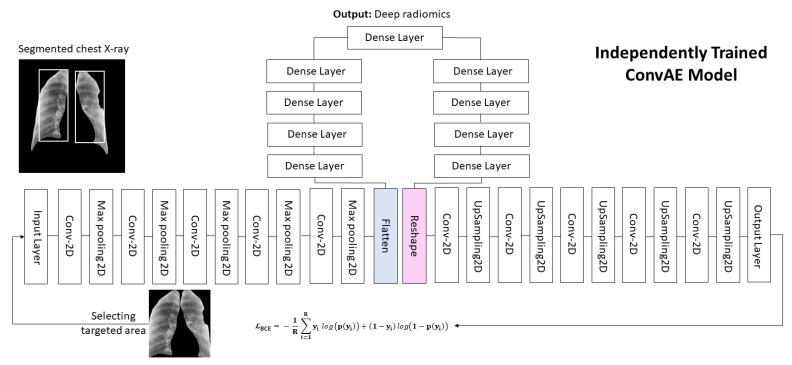
The proposed convolutional deep autoencoder (ConvAE) for generating deep radiomics with low-dimensional deep radiomic descriptors.

The U-Net architecture [39] contains an encoding (contracting) path and decoding (expanding) path in the model. The encoding path shrinks the input spatial dimension to reduce the size, and the decoding pathway enlarges the dimensionality of the data and then generates segmentation maps as the outcome. The initial design does not use padding in convolutional layers, leading to a smaller output segmentation map. However, as we would like to preserve the same initial spatial range of the input and the output, we used padding in the model’s architecture. Our modified architecture had 32 convolutional layers in the encoding (contracting) path. All the CXR images were normalized, and for every convolutional layer, batch normalization and a rectified activation linear unit (ReLu) layer were used. After every consecutive convolutional layer, three 2 × 2 pooling layers decreased the input spatial dimension of 512 × 512 to a reduced dimensionality of 32 × 32 as the outcome of the contracting path (encoder). After that, these compressed data were directed to a decoder (expanding path), which has an encoder with skip connection (bridges) between two paths. This path was deconvolved by upsampling the data with a constant kernel size (2 × 2). The transitional data from the encoder were then appended to the upsampled data in each layer for the entire path, which facilitated the model to reconstruct the information lost throughout the max-pooling process. Our model took a CXR image with a single channel and spatial size of 512 × 512 for 2D and a cube size of 128 × 128 × 64 for 3D model. Figure 2 shows more details about the network architecture. Additionally, the convolutional and deconvolutional layers with their filters are presented in Figure 2, which can help the readers follow changes in data dimensionality through the model. The overall trainable parameters in our network are 7,759,521.

Dice loss: The Dice loss function originated from the Sørensen–Dice similarity coefficient in the 1940s and was used to measure the similarity between two samples [40,41]. The Dice loss is used for 3D segmentation of medical imaging in 2016 [42]. Dice similarity coefficient (DSC)’s definition is represented as follows:

(1) $$\mathrm{DSC}=\frac{2\sum_{i}^{N}p\left(y_{i}\right)g_{i}}{\sum_{i}^{N}p{\left(y_{i}\right)}^{2}+\sum_{i}^{N}g_{i}^{2}}$$

where $p\left(y_{i}\right)$ is the predicted probability value of the segmented label i, and $g_{i}$ represents the actual label value to be segmented by our model. i changes according to the number of segments (here we have two labels lung lobes and background).

**Binary cross-entropy loss function/log loss:** Binary, or sigmoid, cross-entropy (BCE) [43] is a cross-entropy loss function modified by adding a sigmoid activation function in the overall loss as follows:

(2) $${ℒ}_{\mathrm{BCE}}=-\frac{1}{R}\;\overset{R}{\underset{i=1}{\sum }}y_{i\;}log\left(p\left(y_{i}\right)\right)+\left(1-y_{i}\right)log\left(1-p\left(y_{i}\right)\right)$$

where y represents the actual label and p(y) is the predicted probability value of the segmented label for all R points. The use of a sigmoid function, $\frac{1}{1+e^{-y}}$, allows the function’s binarization, representing the existing class against the background class.

### 2.2. Deep Radiomics

Convolutional neural networks (CNNs) are recognized deep neural network structures that have been widely used for a variety of applications, especially in medicine [44]. The convolution layers made by additive adaptive filters that control the receipt field of the layers have increased the popularity and efficiency of the model [45,46,47,48]. We have already used CNN in the U-Net to segment the lung lobes in the previous section. Another known form of utilizing such models is to extract hidden layer weights of a pretrained model and use them as features, called deep radiomics, or deepomics. Some successful pretrained models used for this purpose are AlexNet [45], deep residual network (ResNet) [49], VGG network [50], and GoogleNet (also codenamed Inception_v1) [51]. In medical imaging and, specifically, COVID-19 imaging research, many CNN models were used as pretrained, fine-tuned, or slightly modified models, which showed promising results to detect COVID-19. Pretrained ResNet and InceptionV3 models were used in various versions either individually or combined to detect COVID-19 infection in CXR imaging [10,28]. The transfer learning method was applied to one or a combination of several pre-trained CNN models (i.e., Xception) for detecting COVID-19 [8,10,34]. An application of deep learning to deal with the high dimensionality of deep radiomics using Xception + ResNet50V2 involves convolutional and drop-out layers [34], which has some similarities with a parallel-dilated convolutional neural network architecture [37], despite the fundamental differences that come from using two distinct deep-learning pathways instead of pretrained models. Similarly, there are some other variations of pretrained models labeled with other names (i.e., CoroNet [30] and COVIDX-Net [36]), which achieved considerable performance in differentiating COVID-19 from pneumonia and normal cases. Several deep-learning-interconnected models used a hierarchy of CNN models with more innovative configurations to conduct classification, such as DarkCovidNet [35] and COVID-Net [9].

The extraction of radiomics-based features also has sparked great interest among researchers in the field, in which a large number of features from CXR images are extracted through deep-learning models (e.g., ResNet) and SVM [31] or conventional radiomics approaches [52,53] used to perform computer-aided diagnosis. Although better diagnostic/prognostic decisions are made by having higher dimensional features to capture the characteristics of the medical data better, the abundance of attributes creates a problem called the *curse of dimensionality*. It lowers the accuracy while showing pseudo improvement in the overall accuracy of the system due to the increased collinearity among the features. To alleviate this problem, one potential solution is to use deep-learning feature selection for deep radiomics [54].

In this study, a deep convolutional autoencoder modified with dense layers was used. Several initial layers are convolutional layers with 3 × 3 filter size and dense layers to convert the input images to *latent space* and extract the representation code of the input [52,55]. Such a stacked autoencoder encodes the input CXR image into compressed latent space radiomics, also known as code, within the lower-dimensional space. With these codes, the autoencoder reconstructs the original input similar to decoder path modules [56,57]. The proposed architecture carries out the self-learning representations from CXR images and analyzes the latent space-compressed radiomic features in terms of their discriminative capacity. By training the autoencoder, the model learns how to reconstruct the compact representation of input data.

Here, we propose the CNN-based radiomics through a stacked autoencoder trained exclusively for such high-dimensional input CXR images. Autoencoders are data specific, spontaneously learned from training input CXR images, with a completely independent dataset, instead of achieving by human interference and lossy. This model is trained based on the loss function comparing the original input and decompressed representations. The parameters of the compression (encoding)/decompression (decoding) functions are updated in such a way that they minimize the reconstruction loss using stochastic gradient descent (SGD) [56,57].

Consider a 10-layer convolutional autoencoder model, as depicted graphically in Figure 2. Let $x\in {\mathbb{R}}^{M\times M}$ with M=512  be the CXR input image that passes through the 5-layer convolutional encoder. In each encoder layer, there is a filter bank $K_{f}\in {\mathbb{R}}^{3\times 3}$, with a dilation rate of 1 and fixed f=1,2,…,F, (F = 32) number of filters for the entire model [58]. The convolution layer creates a feature map $Y_{f}\in {\mathbb{R}}^{\overset{´}{M}\times \overset{´}{M}}$, by 2D discrete convolution, and is shown by the following:

$$Y_{f_{i,j}}=\overset{2}{\underset{x=0}{\sum }}\overset{2}{\underset{y=0}{\sum }}K_{f_{2-x,2-y}}X_{1+s\left(i-1\right)-2,1+s\left(j-1\right)-2}\;$$

where $\overset{´}{M}=M$, and s≥1 is called a stride. In general, a single layer model is an affine transformation of x by a nonlinear function $\hat{y}=f\left(Wx+b\right)$, where the weight matrix is $W\in {\mathbb{R}}^{D\times M},\;$ **b**$\;\in {\mathbb{R}}^{D}$ is a bias term, and f(.) is a nonlinear function. The output $h_{p}$ of a layer, p is presented by the following:

(3) $$h_{p+1}=f_{p+1}\left(W_{p}h_{p}+b_{p}\right)=f_{p+1}(W_{p}f_{p}\left(\ldots \left(f_{1}\left(W_{0}x+b_{0}\right)\right)\ldots +b_{p-1})+b_{p}\right)$$

where $h_{1}=f_{1}\left(W_{0}x+b_{0}\right)$ is the first layer’s output, and $h_{p}$ is known as a feature vector at the path layer. The proposed autoencoder consists of a multiple-layer encoder denoted as E.

(4) $$h=f_{E}\left(x;\theta_{E}\right)$$

where h is the output of the encoder or the features (CNN-based radiomics) and $\theta ={\left\{W_{p},b_{p}\right\}}_{p=0}^{p-1}$. A decoder network showed as D also reconstructs the input x by the following:

(5) $$\hat{x}=f_{D}\left(h;\theta_{D}\right)$$

Training this autoencoder then involves calculating the expected reconstruction loss over all the training samples [58] as follows:

(6) $$\theta_{E}^{*},\theta_{D}^{*}=\arg\underset{\theta_{E},\theta_{D}}{\min}\;{𝔼}_{(x,y)∼𝒫}\left[ℒ\left(f\left(x;\theta \right),y\right)\right]$$

where 𝒫 is data generating distribution and $ℒ\left(\hat{y},y\right)$ is discrepancy loss of the reconstructed and input data. We use BCE loss here, as presented in Equation (2). Neural networks are often trained using SGD or its variations [59,60] in which gradients are computed with the backpropagation procedure [61]. The proposed ConvAE model has a 3×3  kernel filter, 2×2 kernel stride, same padding, with no residual connection, and dese layer between contraction and expansion paths (see Figure 2).

### 2.3. Conventional Radiomics Features

Decoding tumor imaging information into subvisual and quantitative features through the extraction of radiomics features as mineable data follows four steps: image acquisition; selection of the region of interest (ROI); extraction of features; decision making (predictive/prognosis method) [62,63,64]. The proposed approach employed 354 radiomics features in 9 families: first-order statistics (FO), shape-based expression (SB), gray level co-occurrence matrix (GLCM), gray level dependence matrix (GLDM), gray level run length matrix (GLRLM), gray level size zone matrix (GLSZM), neighboring gray-tone difference matrix (NGTDM), Laplacian of Gaussian (LOG), and three-layer filtering wavelet. The dimension of these conventional radiomics renders the data analysis prone to the *curse of dimensionality* problem.

This study used three different unsupervised feature selection approaches trained on our training set and independently tested our targeted cohorts. We applied the Johnson–Lindenstrauss (JL) *lemma* [65,66], Laplacian scoring [67], and principal component analysis (PCA) [68] to reduce the dimensionality of conventional radiomics extracted by spanning the current manifold of data onto low-dimensional space using a sparse Achlioptas matrix. All three of these approaches were applied to the Shenzhen set (*n* = 704) in the discovery stage. Then, the entire parameters of the system were frozen for the independent validation step (*n* = 1597). Having our deep and conventional radiomics compressed, we used a random forest to classify COVID-19 patients from pneumonia and healthy cases using each category of the radiomics (i.e., ConvAE latent space, conventional radiomics, or a combination of both) (Figure 1).

## 3. Results

Our models (U-Net and ConvAE) were trained and independently validated with three other datasets to examine the proposed systems. In the next section, we describe all datasets in detail. We used only COVID-19 cases that have confirmed lung manifestation. These datasets were selected randomly and based on having a higher number of COVID-19 patients. Table 1 presents number of patients used for training and validation of the proposed approach.

The resulting U-Net to segment the lung area and ConvAE to extract CNN-based radiomics were subsequently evaluated in terms of the ability to diagnose COVID-19 versus healthy and pneumonia patients.

### 3.1. Study Population

#### 3.1.1. Pulmonary Chest X-ray Disease Detection

**Figure 3:**
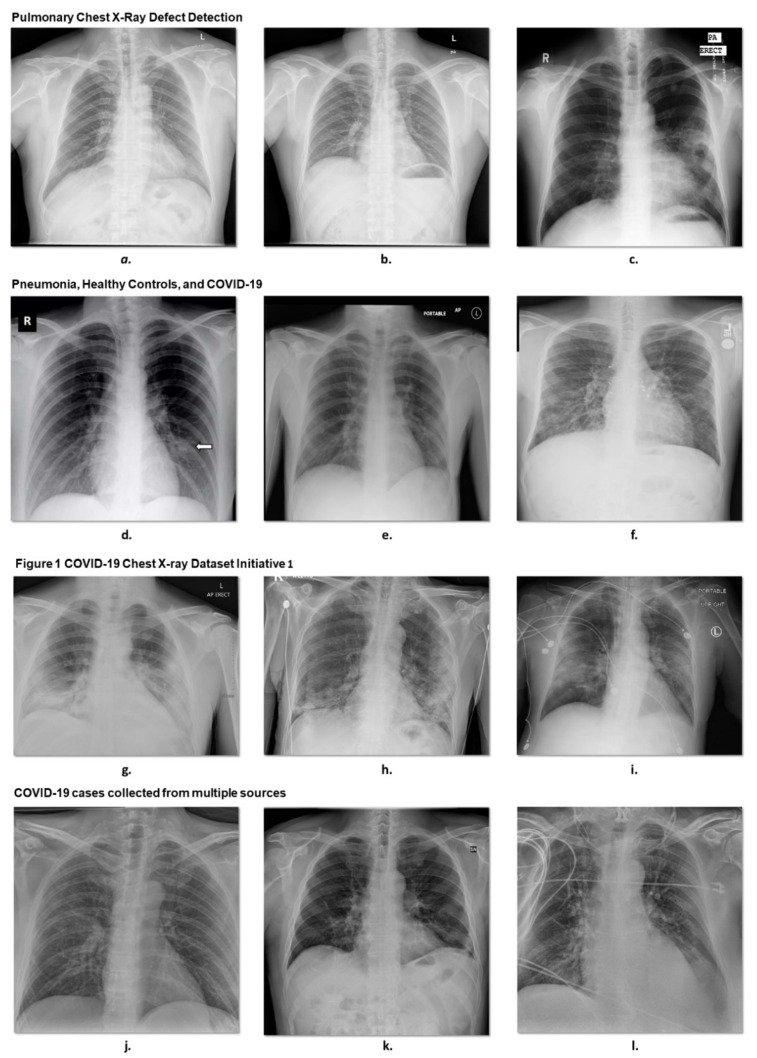
Some examples of CXR images for each dataset are presented: (**a**–**c**) are our 704 cases of training set taken from the Shenzhen set; (**d**–**l**) images are used to independently validate our system.

To design and test the U-Net model for the segmentation of the lung lobes, we used 704 chest X-ray images from the Pulmonary Chest X-Ray Defect Detection, Montgomery County X-ray set, and China set—The Shenzhen set—Chest X-ray Database provided by the National Library of Medicine, National Institutes of Health, Bethesda, MD, USA, and Shenzhen No. 3 People’s Hospital, Guangdong Medical College, Shenzhen, China [69,70]. For the Montgomery County X-ray set, images were acquired from the Department of Health and Human Services (the tuberculosis control program) of Montgomery County, MD, USA. The dataset contains 138 posterior–anterior X-rays, of which 58 cases are abnormal with manifestations of tuberculosis, and 80 cases are normal. The rest of the data were from the Shenzhen set, which contains 336 cases with tuberculosis and 326 normal cases. From the data, we selected 566 cases based on the availability of their masks. Figure 3a–c presents some examples of this dataset.

#### 3.1.2. COVID-19 Cases Collected from Multiple Sources

In total, 417 frontal chest X-rays of COVID-19 cases were selected from the dataset, 761 images with 679 frontal chest and 82 lateral views. Of the 679 frontal images, 518 were standard frontal posteroanterior–anteroposterior (PA/AP) views, and 161 were AP laying down supine all of which were collected from different hospitals across 26 countries. In total, there were 468 COVID-19 (SARSr-CoV-2), 16 SARS (SARSr-CoV-1), 10 MERS-CoV, 5 Varicella, 4 Influenza, 3 Herpes viral cases, 13 *Streptococcus* spp., 9 *Klebsiella* spp., 4 *Escherichia coli*, 4 *Nocardia* spp., 5 *Mycoplasma* spp., 7 *Legionella* spp., 2 Unknown, 1 *Chlamydophila* spp., 1 *Staphylococcus* spp. bacterial cases, 24 *Pneumocystis* spp., 2 *Aspergillosis* spp. fungal cases, 8 Lipoid, 1 Aspiration, and 59 unknown cases in the age of 20 to 90 years old [71,72,73,74,75,76,77,78,79,80] (Figure 3j–l).

#### 3.1.3. Pneumonia, Healthy Controls, and COVID-19

In total, 1125 chest X-ray cases were collected from multiple data sources. Of those, 125 COVID-19 cases were selected from [81], whereas 500 normal and 500 pneumonia cases [35] were selected from the ChestX-ray8 database [80] (Figure 3d–f).

#### 3.1.4. Figure 1 COVID-19 Chest X-ray Dataset Initiative

A total of 55 frontal chest X-ray images of COVID-19 cases were selected from Figure 1 database. DarwinAI and the University of Waterloo have launched an open-source project as a part of the COVIDx dataset to develop the models for COVID-19 detection (COVID-Net) and COVID-19 risk stratification (COVID-RiskNet) [9,82,83,84]. Figure 3g–i shows three examples of this dataset.

All the data above were collected from multiple sources, which are publicly available through online websites, such as Figure 1 [84,85], Radiopaedia.org, the Italian Society of Medical and Interventional Radiology [86], and the Hannover Medical School [85,87]. The data are also accessible in the form of images from online publications, websites, or directly from PDFs using tools, such as pdf images [88]. All the images used for different parts of the proposed approach were frontal X-rays with an overall size of 512 × 512 pixels.

#### 3.1.5. U-Net-Based Segmentation of the Lung Lobes

A total of 704 CXR images were randomly stratified into two training and testing groups, having 566 and 138 cases, respectively. We used the U-Net structure for 2D CHX imaging segmentation [89]. The input CXR images had a 512 × 512 dimension. All input images were normalized, and for every convolutional layer, batch normalization and a rectified activation linear unit (ReLu) layer were used. Every consecutive convolutional layer with a filter size of 3 × 3, 2 × 2 pooling layers decreased the input spatial dimension of 512 × 512 to a smaller dimension of 32 × 32 at the end of the encoder (contracting path). In the convolution layer, 16 filters were convolved with the input CXR images. The model used the same size padding with a 2 × 2 stride. In the decoder (expanding path), the model was a mirrored architecture of the encoder with skip connection (bridges) between two paths, as shown in Figure 1. This path was deconvolved with the upsampled data with a 3 × 3 kernel size. The intermediate data from the encoder was appended to the upsampled data in each layer for the entire path, which helps the model to reconstruct some of the information lost during the max-pooling operation. The overall number of trainable parameters in our network was 7,759,521, and the maximum number of channels was 512. The Adam optimizer trained all models with a modifying learning rate of $2\times 10^{-4}$ to $\;10^{-6}$. The models were trained for 100 epochs with a batch size of 16 for the described cohort of patients. The proposed approach was implemented with the TensorFlow library in Python programming language [90,91] (for training and testing the model). The segmentation results were achieved throughout the inference process by applying the binary accuracy to the predicted similarity with ground truth (GT) labels, which reached close to 98% for the validation set. Then, this trained model was used to segment the lung lobes for other cohorts of patients and generated masks for 1597 CXR images preparing the data for extracting radiomics.

#### 3.1.6. Conventional and Deep Latent Space Radiomics

**Figure A1:**
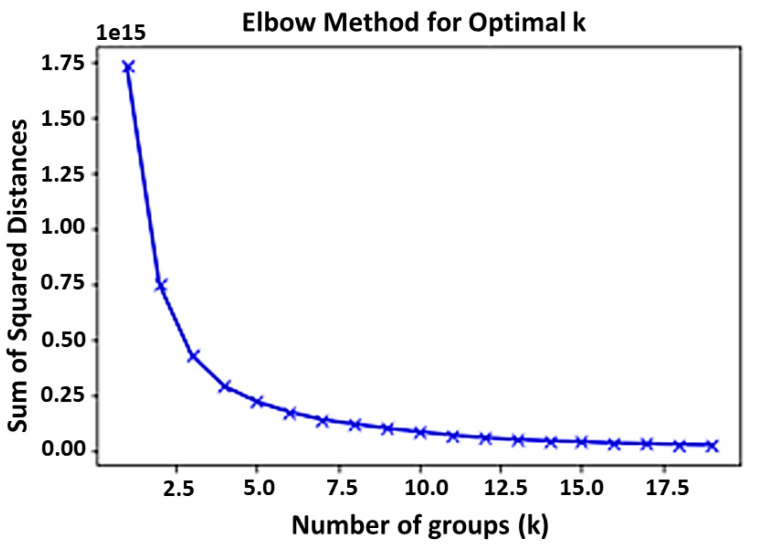
The elbow method to find the optimum cluster number points out k = 7 as the best number of features selected to represent collinearity.

We extracted 354 conventional radiomics [64] using the original CXR images, and the U-Net obtained their corresponding masks for the validation sets. These datasets were used to extract radiomics for their 2D targeted ROI (i.e., solely the lung lobes area) using the trained model. Out of all conventional radiomics, seven descriptors were selected using three different JL, LS, and PCA approaches. These seven descriptors were chosen based on the elbow method of choosing the best number of grouping features (Figure A1 in Appendix A).

Our proposed ConvAE model comprised 5 convolutional blocks and had 636,929 trainable parameters, with a preferred signal channel input image dimension of 512 × 512. The input image then passed through 16 filters with 3 × 3 kernels. The rescaling dimensions of the input image are from 512, 256, 128, 64, 32, and 16, while the fourth dimension grew from 1, 16, 32, 64, and 128. In the middle of the model, the hierarchy of dense layers compressed the dimensionality from 16,384 to 16, which generated 16 deep latent space features. The proposed ConvAE model has a 3×3  kernel filter, 2×2 kernel stride, same padding, with no residual connection with batch normalization and dese layer between contraction and expansion paths (see Figure 2). Out of 16, 2 features (FC-8 and FC-16) were discarded due to zero, and 14 other features were used for classification.

**Figure 4:**
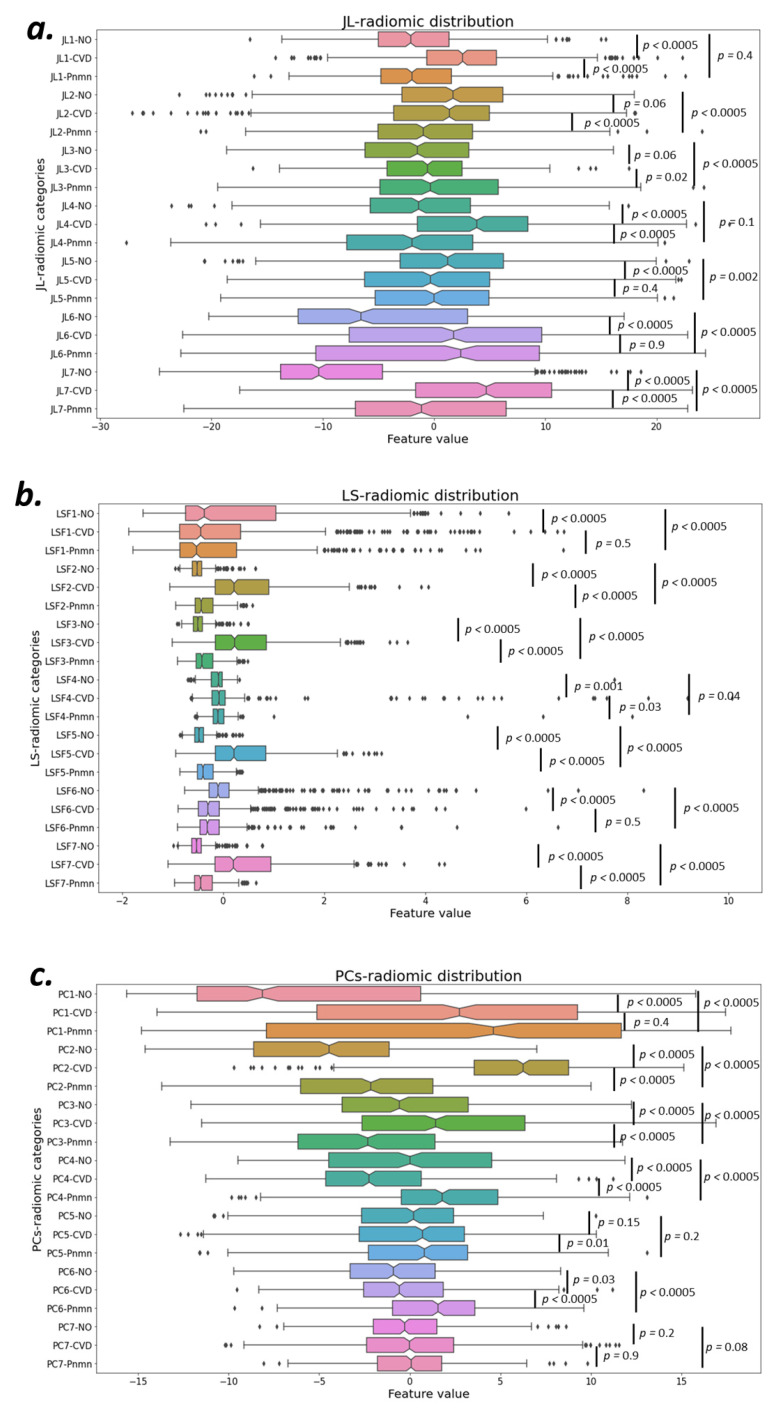
Classification power of each selected radiomic group to categorize COVID-19 (CVD), pneumonia (Pnmn), and no finding (NO) cases with respect to ground truth are demonstrated using Kruskal–Wallis test to show the statistical significance of the classification of conventional radiomics using three dimensionality reduction methods JL (**a**), LS (**b**), and PCA (**c**).

**Figure 5:**
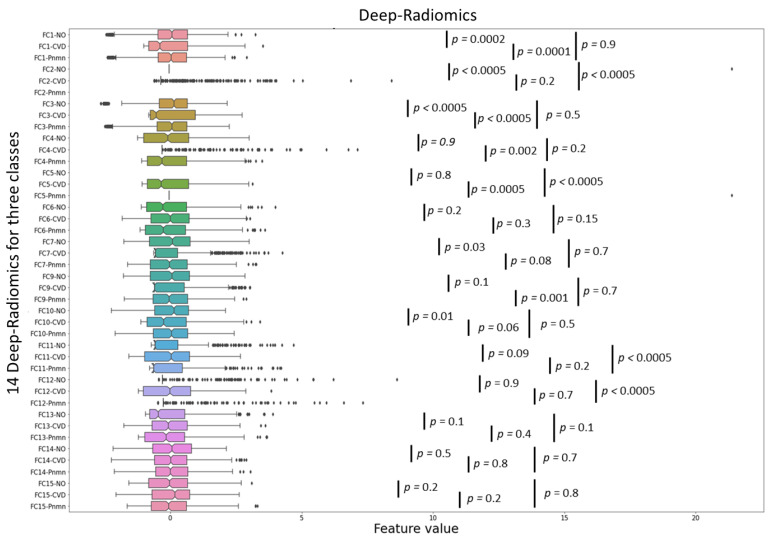
Similar to conventional radiomics, here classification power of 14 deep radiomic groups to categorize COVID-19 (CVD), pneumonia (Pnmn), and no finding (NO) cases with respect to ground truth are demonstrated using Kruskal–Wallis test to show the statistical significance of the classification.

Figure 4 presents the separation power of each selected conventional radiomics to categorize COVID-19 (CVD), pneumonia (Pnmn), and no finding (NO) cases concerning the GT using the Kruskal–Wallis test to show the statistical significance. Similarly, Figure 5 shows the classification of 14 deep-radiomics obtained from the ConvAE model. The separation among different groups was more statistically significant for PCA (Figure 4a) and LS (Figure 4b). However, JL and deep radiomics also showed reasonable separation between different cases. For instance, FC2 responds only for COVID-19 cases and no response for pneumonia and healthy cases.

### 3.2. Random Forest Classification of COVID-19

To calculate the quantitative accuracy, we employed clinical diagnosis as the GT for our calculation. The GTs were labeled as 1 for COVID-19, 2 for pneumonia, and 0 for healthy cases. In binary-class classification, we labeled COVID-19 as 1 and the other two groups as 0.

**Figure 6:**
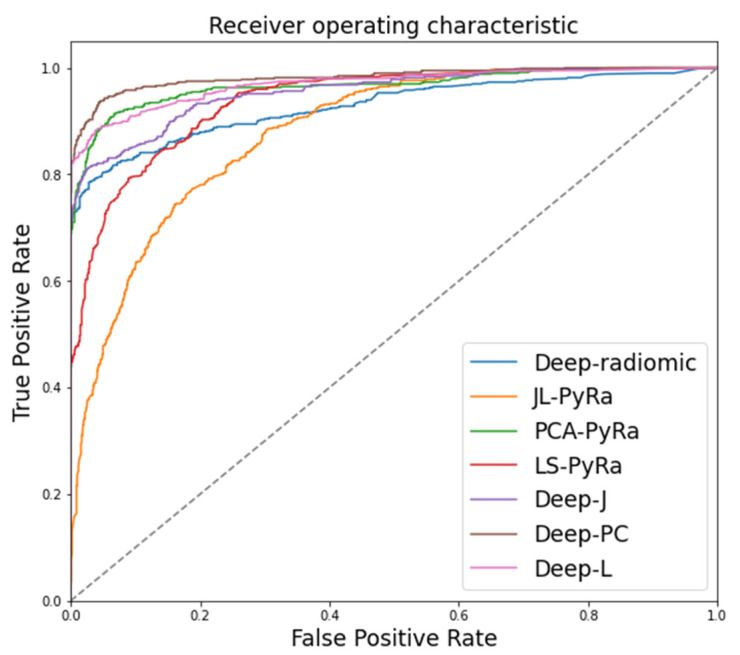
The receiver operating characteristic (ROC) curves for deep radiomics and compressed conventional radiomics using JL, LS, and PCA, and a combination of both deep and conventional radiomics are shown.

We created three categories of patients based on imaging biomarkers to perform classification using a random forest classifier. Three imaging biomarkers included conventional radiomics, deep radiomics, and a combination of both groups. We classified 1597 cases based on 14 ConAE deep latent space radiomic descriptors, 7 conventional radiomics, and finally, a combination of both while comparing them with the gold standard data (GT) from clinical assessment. To investigate which type of imaging biomarkers is more appropriate for classifying COVID-19 patients, we performed both binary and multi-class classification and trained our multivariate random forest classifier with leave-one-out cross-validation in which we randomly split the data into training and testing sets. Table 2 and Figure 6 show the cross-validated accuracy of each method and their receiver operating characteristic (ROC) curves.

The best multivariate binary-class classification model for COVID-19 versus non-COVID-19 (i.e., pneumonia and no-finding) cases was PCA, which resulted in an accuracy of 89.6% (88.4–90.7%), and deep-LS and deep-PCA for the combination of both radiomic types (conventional radiomics and deep radiomics) resulted in 88.9% (88.7–93.2%, and 88.8% (88.7–89.0%), respectively.

**Figure 7:**
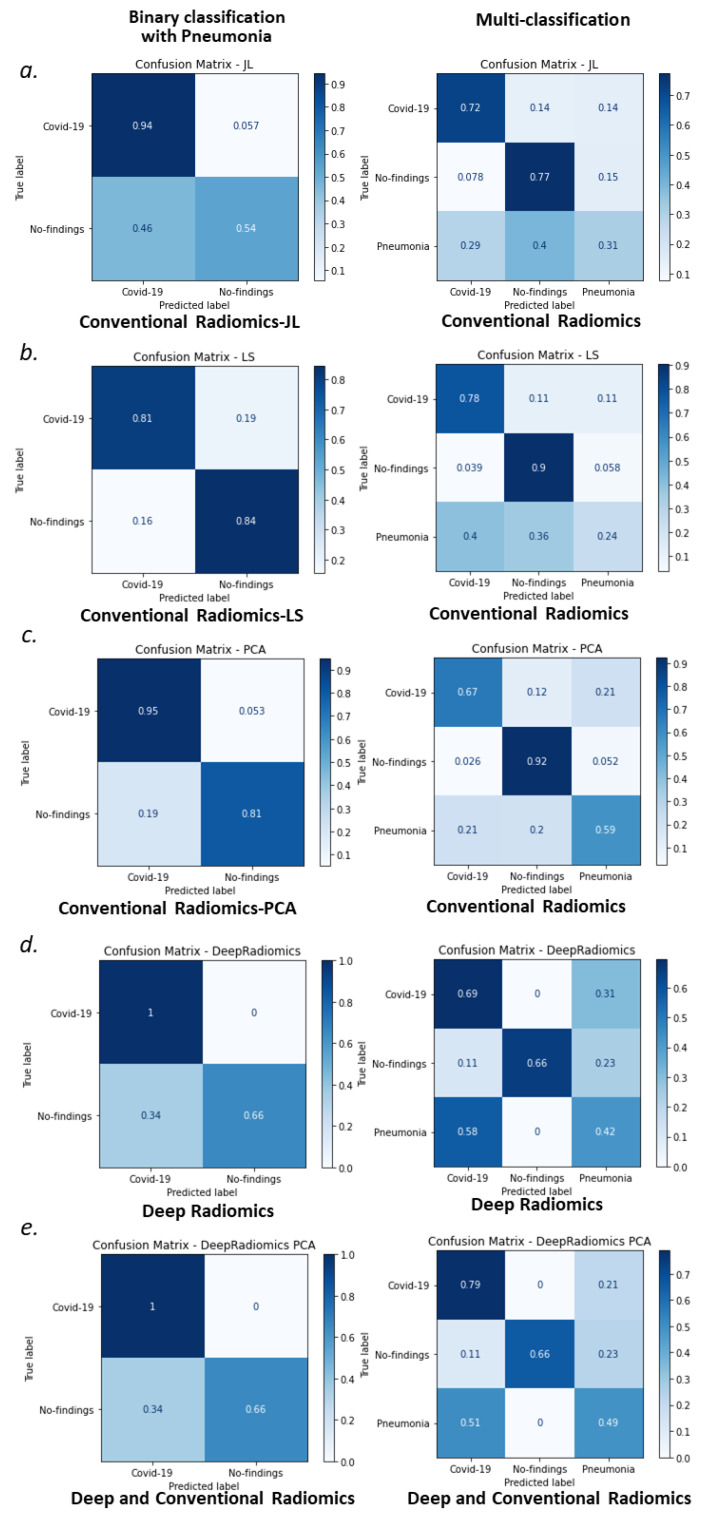
Confusion matrices for binary-class (upper row) and multiclass (lower row) classification of COVID-19 are presented for conventional radiomics (**a**–**c**); deep radiomics (**d**), and a combination of both groups of radiomics for the best conventional radiomics (**e**).

JL radiomics showed the lowest binary-class classification accuracy 79.3% (77.2–80.3%) to detect COVID-19 versus other cases. This might be due to spanning features onto a lower-dimensional space that preserves the Euclidean distance between features but not selecting the most effective radiomics, increasing the accuracy of classification. This trend is also observed in multiclass classification and JL 63.9% (62.5–65.2%), which showed the lowest classification accuracy after deep radiomics, 63.5% (61.3–65.1%). PCA alone showed the highest accuracy for both binary 89.6 (88.4–90.7) and multiclass 72.6 (69.4–74.4) classification. Once combined with deep radiomics, PCA resulted in 88.8 (88.7–89.0) and 72.5 (71.2–73.4) for binary and multiclass classifications, respectively. Deep-LS showed the lowest accuracy, 66.8% (65.9–73.4%), in multiclass COVID-19 classification when both types of features are combined. We also calculated the statistical difference of the maximal accuracy (PCA) with other approaches using a two-tailed *t*-test (see Table 2). Deep-LS showed considerable similarity to PCA covariates (*t*-statistic = 1.05, *p*-value = 0.29), and there was a marginal statistical significance for deep-PCA (*t*-statistic = 2.3, *p*-value = 0.02). The rest of the methods showed significant statistical differences. Figure 6 shows the confusion matrices for binary- and multiclass classification of COVID-19. For binary-class classification, we stratified patients into COVID-19 cases versus “other” cases (i.e., no finding and pneumonia). Among the five groups, conventional radiomic PCA (Figure 7c—left column), deep radiomics (Figure 7d—left column), and their combination (Figure 7e—left column) showed the highest accuracy of COVID-19 detection. In multiclass classification, LS radiomics (Figure 7b—right column) and deep-radiomics-PCA (Figure 7e—right column) showed the highest accuracy of detecting COVID-19. PCA-radiomics (Figure 7c—right column) showed the highest accuracy of finding healthy and Pneumonia cases.

The application of deep ConvAE created deep radiomics and provided a low-dimensional representation of ROI. It also demonstrated a substantial increase in model performance for classifying COVID-19 patients from healthy or pneumonia patients (Figure 6, and Table 2). Deep radiomics is known for its tendency of having high dimensionality, thereby intensifying the possibility of overfitting a decision-making unit (i.e., the random forest model in this study) and the *curse of dimensionality* problem. The proposed ConvAE provided low-dimensional deep radiomics by spanning the imaging features to a lower-dimensional space [54]. We applied dimensionality reduction for the conventional radiomics by following the traditional way to shrink features while preserving the image characteristics. Despite having a different structure and mechanism, our proposed model is comparable in terms of the accuracy of COVID-19 detection. It shows a slight difference from previously reported models such as CoroNet [32] (89.6% accuracy), COVID-Net (93.3% accuracy), ResNet-50 (90.6% accuracy), and VGG-19 (83.0% accuracy) [83].

## 4. Discussion

This study proposed an automated system to diagnose COVID-19 using standard and deep radiomics in chest X-ray imaging. Specifically, this study demonstrated a deep-learning-based dimensionality reduction model, which integrates with selected conventional radiomics for diagnostic purposes. We showed the possibility of independently validating two deep-learning models to segment the lung lobe areas and identify potential COVID-19 patients enabling fast, noninvasive, and more cost-effective CXR-imaging-based diagnosis of COVID-19. The training of our models was absolutely independent of our training cohorts of patients. These datasets are selected randomly and based on having a higher number of COVID-19 patients. Overall, the study impartially validated with approximately the same size patients’ cohorts (597: COVID-19 and 500: Pneumonia patients, and 500 healthy cases), which increased the possibility of fair and unbiased assessment.

One limitation of the presented approach is the lack of clinical information in our data. Even with the substantial number of cases used for model development, there is a need for the models to incorporate clinical input to develop a more reliable diagnostic system. Additionally, there is a need for more data from various sources to further validate the present work and assess the model’s generalizability, or association to some demographics and clinical factors such as race (similar to [92]). Although our data are relatively large, there are only three label categories: COVID-19, pneumonia, and healthy. Having a more significant cohort of patients with more categories will enhance the statistical power of our analysis by improving the benchmarking system. Furthermore, there are more configurations for extracting deep radiomics. These configurations may allow us to find a better compression scheme that captures deep-imaging biomarkers with superior discriminative capability, efficiently representing the disease characteristics in CXR imaging. For conventional radiomics, the current random projection of high-dimensional manifold to low-dimensional representative radiomic signatures can be made through a more established, selective, and systematic approach. There is also an option to make diagnostic decisions directly using the trained deep learning model instead of using it for deep radiomic extraction. However, this would involve different configurations for training the model.

The techniques presented in the current study offer several advantages. First, applying ConvAE to extract deep radiomics provides an effective way of projecting CXR imaging biomarkers to lower-dimensional radiomic signatures, which avoids pretrained models with higher dimensional features and overfitting the decision-making unit. Second, the ConvAE eliminates the human-engineering feature selection to decrease the dimensionality of deep radiomics. Third, the proposed method successfully showed good performance on the independent validation sets for lung lobe segmentation and generation of deep radiomics.

## 5. Conclusions

This study proposed an automatic diagnosis of COVID-19 in CXR imaging using deep and conventional radiomics features. A 2D U-Net model was trained to segment the lung lobes, and from these regions of interest, radiomic features were extracted. We performed dimensionality reduction using a convolutional deep autoencoder (ConvAE) to extract low-dimensional deep radiomics (14 features) and Pyradiomics library to extract conventional imaging biomarkers. Johnson–Lindenstrauss (JL) *lemma*, Laplacian scoring, and principal component analysis (PCA) were used to reduce the dimensionality of features from 354 to 7 radiomics for conventional radiomics. We trained the entire system using 704 CXR images then independently tested our system on 1597 patients with COVID-19, pneumonia, or no finding. We compared the performance of each type of radiomic feature to detect COVID-19 cases through multivariate binary-class and multiclass classification. We trained and tested a random forest model for detecting COVID-19 cases through multivariate binary-class and multiclass classification. The maximal (full multivariate) model using a combination of the two radiomic groups showed excellent performance in classifying cases with a cross-validated accuracy of 72.5 (71.2–73.4) for multi-class and 88.8 (88.7–89.0) for binary-class classification. For future work, other deep-learning-based configurations can be explored to increase the system’s overall accuracy.

## Figures and Tables

**Table 1:** Training and validation scheme for the proposed approach.

Scheme of Model Training and Independent Validation			
Purpose	Model	Learning Stage	Number of Cases and Dataset
Segmentation of Lung Lobes	U-Net ^1^	Training	704 The Shenzhen set
		Independent Validation	417 COVID-19
			1125 ChestX-ray8
			55 Figure 1
COVID-19 Identification	ConvAE ^2^	Training	704 The Shenzhen set
		Independent Validation	417 COVID-19
			1125 ChestX-ray8
			55 Figure 1

^1^ This is a 2D U-Net model. ^2^ ConvAE was designed and trained on our training cohort of patients to generate CNN-based radiomics (i.e., deepomics).

**Table 2:** Results of diagnosis based on the classification of symptomatic versus asymptomatic patients with the leave-one-out cross-validation.

Accuracy of Multivariate Models			
Methods		Classification Accuracy ^1^ (%)	*t*-Test ^2^; *t*-Statistic, Two-Tailed *p*-Value
**Binary Classification**	Deep-radiomics	88.7 (88.7–88.9)	8.6, <0.0005
	JL-PyRad	79.3 (77.2–80.3)	>90, <0.0005
	PCA-PyRad	89.6 (88.4–90.7)	-
	LS-PyRad	83.6 (82.9–84.3)	55.4, <0.0005
	Deep-JL	88.7 (88.7–88.8)	8.6, <0.0005
	Deep-PCA	88.8 (88.7–89.0)	7.9, <0.0005
	Deep-LS	88.9 (88.7–93.2)	1.05, 0.29
**Multiclass Classification**	Deep-radiomics	63.5 (61.3–65.1)	>90, <0.0005
	JL-PyRad	63.9 (62.5–65.2)	>90, <0.0005
	PCA-PyRad	72.6 (69.4–74.4)	-
	LS-PyRad	65.7 (65.1–66.2)	72.6, <0.0005
	Deep-JL	67.7 (64.9–71.4)	42.1, <0.0005
	Deep-PCA	72.5 (71.2–73.4)	2.3, 0.02
	Deep-LS	66.8 (65.9–73.4)	32.3, <0.0005

^1^ Classification accuracy reported by median (±QR). ^2^ *t*-test calculated for each method versus maximal accuracy.

## Data Availability

In this study, we used publicly available datasets [69,70,71,72,73,74,75,76,77,78,79,80,81,82,83,84,85,86,87,88,89].

## Appendix A

The Elbow approach has been established to determine the optimum number of features to alleviate collinearity. As shown in the graph (Figure A1), the point for selecting is less than 10% variance of the graph. This indicates somewhere around k = 7. We used this analogy for our feature selection approaches in this study and picked 7 radiomics out of 354 conventional radiomics.
